# Supplementary material for: A Portable Biosensor Based on Au Nanoflower Interface Combined with Electrochemical Immunochromatography for POC Detection of Prostate-Specific Antigen
Source: Biosensors (Basel). 2022 Apr 19;12(5):259. doi: 10.3390/bios12050259 (PMC9138250; doi:10.3390/bios12050259)
Supplement: Supplementary file 1 [file biosensors-12-00259-s001.zip › biosensors-1665772-supplementary.pdf]

biosensors

Supplementary

A Portable Biosensor Based on Au Nanoflower Interface Combined with Electrochemical Immunochromatography for POC Detection of Prostate-Specific Antigen

Yanzhi Dou [1,2], Zhenhua Li [1,2], Jing Su [3] and Shiping Song [1,4,*]

[1] Division of Physical Biology, CAS Key Laboratory of Interfacial Physics and Technology, Shanghai Institute of Applied Physics, Chinese Academy of Sciences, Shanghai 201800, China; douyanzhi@sinap.ac.cn (Y.D.); lzh@sinap.ac.cn (Z.L.)
[2] University of Chinese Academy of Sciences, Beijing 100049, China
[3] State Key Laboratory of Oncogenes and Related Genes, Institute for Personalized Medicine, School of Bi medical Engineering, Shanghai Jiao Tong University, Shanghai 200030, China; sujing@sjtu.edu.cn
[4] The Interdisciplinary Research Center, Shanghai Synchrotron Radiation Facility, Zhangjiang Laboratory, Shanghai Advanced Research Institute, Chinese Academy of Sciences, Shanghai 201210, China
* Correspondence: songshiping@sinap.ac.cn

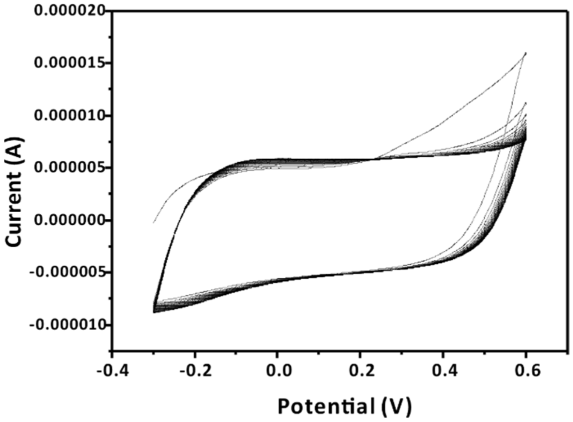

Figure S1. CV curve of SPCE cleaning.

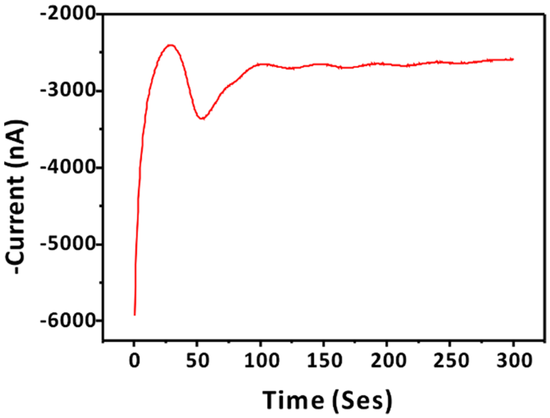

Figure S2. i-t curve of electrochemically deposited Au NFs on the SPCE.
